# The effect of controlled ovarian hyperstimulation on ovarian reserve via PTEN pathway

**DOI:** 10.1530/RAF-21-0075

**Published:** 2022-08-15

**Authors:** Perihan Sezginer, Cigdem Elmas, Fatma Yıldız

**Affiliations:** 1 Department of Medical Laboratory Techniques, Alanya Alaaddin Keykubat University, Health Services Vocational School, Alanya, Turkey; 2 Department of Histology and Embryology, Gazi University, Faculty of Medicine, Ankara, Turkey

**Keywords:** controlled ovarian hyperstimulation, PTEN, FOXO3, LH-R, ovarian reserve

## Abstract

**Graphical abstract:**

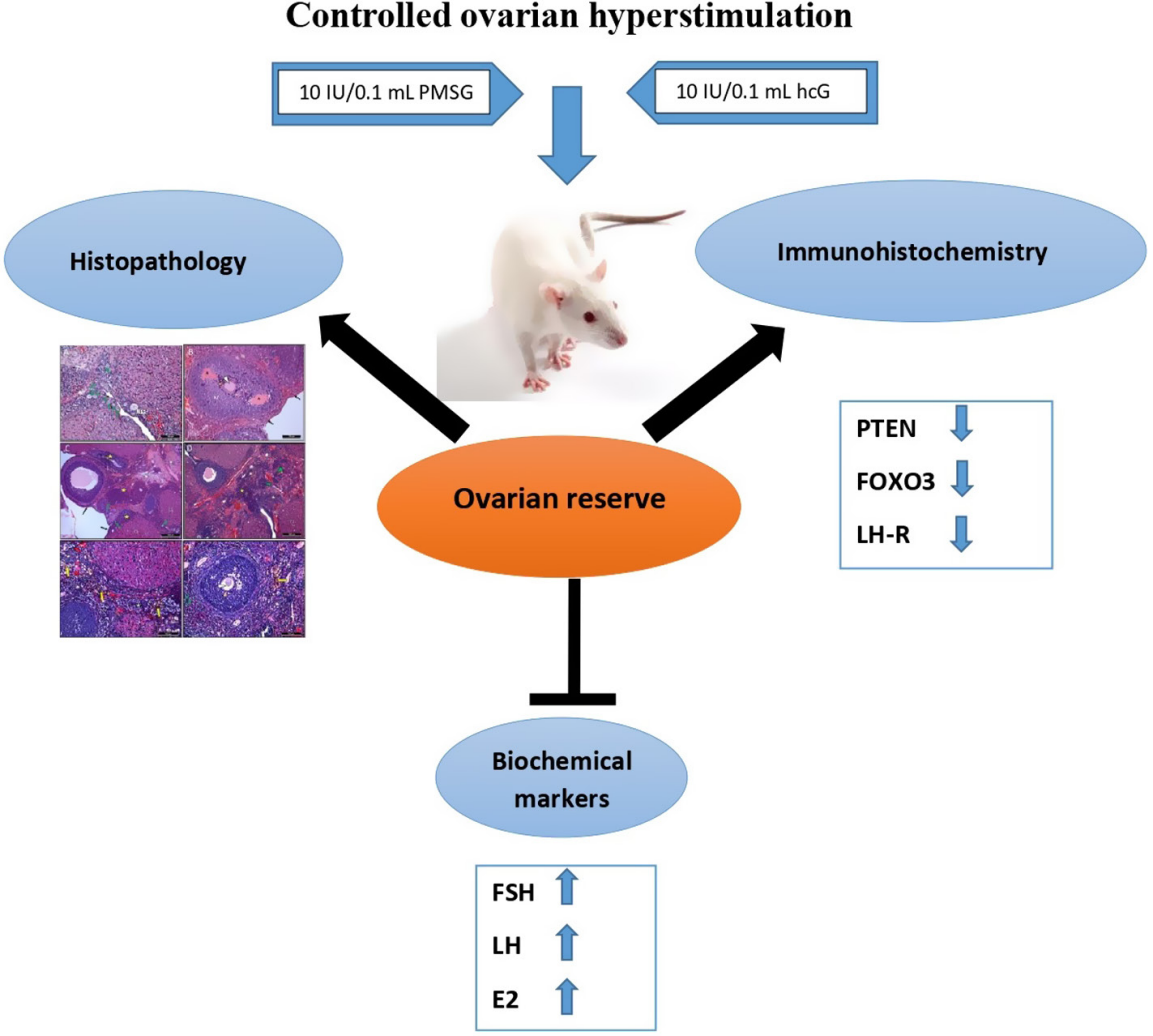

**Abstract:**

This study was carried out to investigate whether repeated controlled ovarian hyperstimulation (COH) affects ovarian reserve. For this reason, we aimed to show possible changes in the expression of PTEN and FOXO3, which are involved in preserving the over-reserve, after applying the COH protocol methods. For this purpose, 18 young *Wistar albino* female rats (8 weeks old) were randomly assigned as group 1 (control), group 2, and group 3 as 6 subjects in each group. Experimental groups were treated with 10 IU/0.1 mL pregnant mare’s serum gonadotropin and a COH protocol consisting of 10 IU/0.1 mL human chorionic gonadotropin injection after 48 h. This procedure was applied three and five times to group 2 and group 3, respectively. For the control groups, the same procedures were performed with 0.1 mL of 0.9% sodium chloride solution. At the end of the experiment, the ovarium tissues were placed in a 10% neutral formaldehyde solution for light microscopic examinations. In histological sections stained with hematoxylin and eosin, the number of ovarian follicles was determined using the physical dissector method. However, the expression of PTEN, FOXO3, and LH-R molecules was evaluated by immunohistochemical methods. As a result of our study, it was concluded that COH administration reduces the expression levels of PTEN and FOXO3 proteins and LH-R, which are among the essential components of the PIK3 intracellular signaling pathway and also increased the levels of hormones such as follicle-stimulating hormone, estradiol, and luteinizing hormone, which are over-reserve markers, and causes adverse effects on the histological structure, oocyte morphology, and number of ovaries.

**Lay summary:**

Today, approximately 10–15% of couples experience fertility problems. However, assisted reproductive techniques help people with fertility problems to get pregnant. The main purpose of these techniques is to put the sperm and egg together outside the woman’s body where the eggs are fertilized and then to return the fertilized eggs (embryos) to the womb. During a woman’s menstrual cycle, several hormones influence the growth of the eggs. This process can be mimicked by using various medications. Medication is given to increase the number of eggs that develop. However, this method is not the same as normal ovulation. Therefore, in our study, we wanted to examine the effect that developing multiple follicles has on the number and quality of eggs remaining for the future.

## Introduction

Controlled ovarian hyperstimulation (COH) is the technique of developing a large number of follicles in the same cycle to obtain the most ideal number and quality of oocytes from the ovaries in assisted reproductive techniques (ART). COH has become an indispensable part of ART in our present day with the increase in infertility cases (Siristatidis & Bhattacharya 2007). For this reason, the effects of hyperstimulation on the human body have recently attracted the attention of many researchers. One of the most important parameters that should be considered in ART is ovarian reserve. The activation of primordial follicles, which represent the ovarian reserve, is under the control of suppressive and activating factors (Adhikari *et al.* 2009, 2010). One of the intracellular signaling pathways that involve these factors is the PI3K signaling pathway. An irregularity in the expression of PTEN, which is one of the main components of the PI3K signaling pathway and which is considered to be a potential protector of the ovarian reserve, causes the primordial follicle pool to be activated earlier. Such a rapid and massive activation of all primordial follicles inevitably ends up in the premature depletion of the follicle pool and results in premature ovarian failure and premature menopause (Goto *et al.* 2007, Ouyang *et al.* 2013, Wang *et al.* 2016). Despite the developments in ART, most patients require multiple COH administrations to achieve pregnancy. This has raised the question of whether repeated COH applications will affect the ovarian reserve of the patient. For this reason, in our study, our purpose was to show possible changes in the expression of PTEN and FOXO3A, which act as suppressor molecules in the transition from primordial follicle to the primary follicle, after COH protocol methods were applied.

## Materials and methods

### Animals and experimental design

The whole experimental study was conducted with the permission numbered G.Ü.ET-17.027/2017 issued by Gazi University, Animal Experiments Local Ethics Committee. In our study, 18 female adult (8 weeks old) *Wistar albino* rats were obtained from Animal Breeding and Experimental Research Center (GÜDAM), Gazi University Laboratory. All animals were maintained with a 12 h light/12 h darkness schedule in the laboratory at a controlled temperature of 23 ± 2°C and were fed with a standard rat diet (Korkuteli Food Industry, Turkey) *ad libitum*. Rats that had normal estrus cycles after vaginal cytologic examination were included in the study (Marcondes *et al.* 2002). The experimental animals were divided into three different groups by randomly selecting six rats for each group.

Group 1: Control group (*n* = 6)
Group 2: Experimental group with three repetitions of COH (*n* = 6)
Group 3: Experimental group with five repetitions of COH (*n* = 6)

### Preparation and administration of gonadotropins

To stimulate folliculogenesis, in the experimental group, follicle-stimulating hormone (FSH) analog pregnant mare’s serum gonadotropin (PMSG, Cat: HOR-272, Prospec, 5 IU/0.1 mL) was injected, and 48 h after this injection, human chorionic gonadotropin (hCG, Cat: C1063-1VL, Sigma, 5 IU/0.1 mL) was administered with i.p injections. PMSG and hCG were dissolved in 100 IU/mL in sterile water for injection before each administration (Quintana *et al.* 2008, Liang *et al.* 2009). This procedure was repeated at 1-week intervals, 3 and 5 times to group 2 and group 3, respectively (Xie *et al.* 2016). The rats in the control group were given 0.1 mL 0.9% NaCl solution instead of PMSG to experience the same stress, and after 48 hours, 0.1 mL 0.9% NaCl solution was given instead of hCG. All rats were sacrificed 1 week after the last injection. At the end of the experiment, all animals were sacrificed under ketamine (45 mg/kg, i.m.) and xylazine (5 mg/kg, i.m.) anesthesia. The ovaries and gonadal fat pads were weighed. After dry weights of the ovary tissues were measured, tissues were first fixed for 72 h in a 10% neutral formaldehyde solution for light microscopic examination, and paraffin blocks were obtained using routine procedures.

### Histological analyses of ovarian tissues

Four-micrometer-thick sections were acquired from the ovarian paraffin blocks of all groups. The ovarian full-thickness sections were prepared for hematoxylin and eosin (H&E) and immunohistochemical (IHC) staining. Every one out of four ovarian sections was used for follicular counting after being stained with H&E with the previously reported method (Kalem *et al.* 2018). IHC staining was performed on ovarian sections with PTEN, FOXO, and LH-R antibodies. Briefly, six zones (one central and five peripheral) were determined randomly from each ovarian tissue sections (5 μm thickness). In these zones, the density of immunoreactivity and the specific pathological criteria of the follicular structures were evaluated under the light microscope.

### Morphological classification of ovarian follicles

The sections were stained with H&E, and follicles were counted based on the following criteria: ‘Primordial follicles’ were defined as an oocyte surrounded by a layer of squamous granulosa cells. ‘Unilaminar primary follicles’ were defined as an oocyte surrounded by a single layer of cuboidal granulosa cells. ‘Multilayer primary follicles’ possessed more than one layer of cuboidal granulosa cells, with no visible antrum, and finally, ‘antral follicles’ contain a clearly defined antral space. In addition, the oocyte-free structures which had a large antral cavity and usually possessed a single layer of cuboidal granulosa cells were defined as cystic follicles (Cheong *et al.* 2014).

### Determination of primordial and primary follicular numbers in the ovary

In our study, the numbers of primordial and primary follicles were determined by using a physical dissector method according to Myers *et al.* (2004) and Altunkaynak *et al.* (2016).

### IHC staining for PTEN, FOXO, and LH-R

Four-micrometer-thick ovarian sections were incubated at 37°C overnight and then at 57°C for 1 h to facilitate deparaffinization. To complete the deparaffinization process, the sections were incubated in xylol twice for 20 min. Then, the sections were dehydrated by incubation in a graded ethanol series (100, 96, 80, 70, and 60%) for 3 min each and then washed with distilled water twice for 5 min each. Sections were fixed in citrate buffer (pH 6.0) (Cat: AP-9003-500, Lot: BBI120127, Lab Vision, Fremont, CA, USA) in a microwave at 750 W for 10 min to ensure that the receptor sites are uncovered from formaldehyde. After cooling to 25°C, the sections were washed with distilled water twice for 5 min each. Tissues on cross sections were circled with a Pap pen. The tissues were then washed with PBS(Cat: AP-9009-10, Lab Vision, Thermo Scientific) (pH 7.4) 3 times for 3 min each followed by treatment with 3% H_2_O_2_ for 15 min (Cat: TA-125-HP, Lot: HP41515, Lab Vision) to block the endogenous peroxidase activity. Next, slides that were washed with PBS three times were treated with Ultra-V block (Cat: TA-125-UB, Lot: AUB161208AI, Lab Vision, Thermo Scientific) to prevent any unwanted interactions. The tissues were then subjected to either PTEN (Cat: ab3139, Lot: GR62840-19, Abcam), FOXO3 (Cat: ab23683, Lot: GR314636-1, Abcam), or LH-R (Cat: bs-6431R, Lot: AD080753, Bioss) primary antibody at 4°C overnight. After incubation, slides (washed with PBS) were treated with a biotinylated secondary antibody.

Slides (washed with PBS) were then treated with streptavidin peroxidase for 20 min to achieve enzyme and biotin link. Finally, nuclear staining was achieved using DAPI. Hematoxylin was used as background staining, and the slides were closed using Entellan. Images were taken on a Leica DCM 4000 computer-aided imaging system and evaluated in Leica Q Vin 3 program.

### Evaluation of PTEN, FOXO, and LH-R expressions in ovarian sections

PTEN, FOXO, and LH-R expressions were scored using an immune reactive scoring scale and evaluated by two researchers who did not have any prior knowledge of the groups of rats. Accordingly, six zones (one central and five peripheral) were selected from ovarian tissue sections (5 μm thickness) and subjected to IHC staining with PTEN, FOXO, and LH-R antibodies. The HSCORE, defined later, was used to evaluate the immune reactive density in these zones. The HSCORE was determined by the following formula: HSCORE ¼ Pi (i + 1), where ‘i’ is the intensity of labeling with a value of 0, 1, 2, or 3 (none, weak, moderate, or strong) and Pi is the percentage of labeled cells for each intensity, within a range of 0–100%. The rate of positive cells was scored by the extent of immunostaining and was assigned to one of the following categories: 0 (0%, no positive cells), 1 (≤30% positive cells), 2 (30–60% positive cells), and 3 (>60% positive cells) (Ishibashi *et al.* 2003).

### Hormonal analysis

Blood samples were drawn intracardially into plain tubes without heparin for endocrine and biochemical analyses, and sera were separated by centrifugation at 1000 ***g*** + 4°C for 10 min. Separated serum samples were placed in polypropylene tubes in small portions and stored at −80°C until analysis.

### ELISA method

Serum FSH, luteinizing hormone (LH), and estradiol (E2) levels were estimated by ELISA. FSH, LH, and E2 analyses were performed using serum FSH (Cat: E-EL-R0391, Lot: AK0017SEP22050, Elabscience Biotechnology Inc., Houston, TX, USA), LH (Cat: E-EL-R0026, Lot: AK0017SEP18050, Elabscience Biotechnology Inc., USA), and E2 (Cat: E-EL-R0065, Lot: AK0017SEP18051, Elabscience Biotechnology Inc., USA) rat ELISA kit. Investigators who were blinded to the sample groups performed all analyses.

### Statistical analyses

The agreement of the continuous variables with normal distribution was examined graphically and by using the Shapiro–Wilk test. Mean ± s.d., minimum and maximum values, and median (interquartile range) values were used to identify the changes. The variables that met the parametric test assumptions (i.e. primary follicle, Graafian follicle, and cystic structures) were evaluated with the one-way ANOVA. When a difference was detected in the ANOVA result, the source of this difference was investigated by using the Bonferroni* post hoc* test. The Kruskal–Wallis test was used to compare the non-parametric variables between the groups (primordial follicle and antral follicle counts, and pTEN, FOXO3, and LH-R expressions). When a difference was detected, *post hoc* pairwise comparisons were made with the Mann–Whitney test and Bonferroni correction to identify the different groups. The statistical analysis and calculations were performed with IBM SPSS Statistics 21 (IBM). In statistical terms, *P* ≤ 0.05 was taken as an indicator of significant difference.

## Results

### H&E staining findings

**Figure 1:**
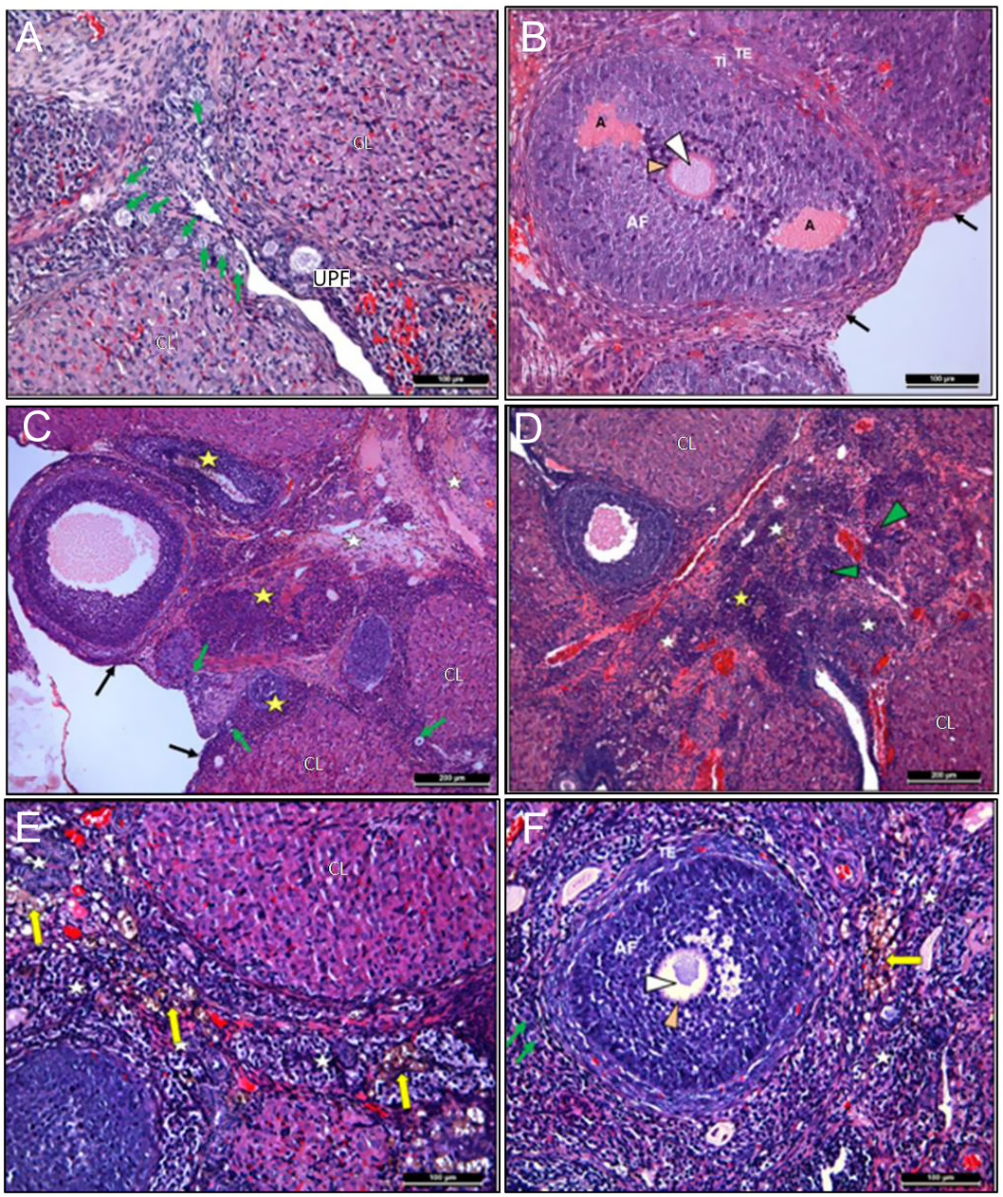
Results of H&E staining of ovarian tissues. Primordial follicles (green arrows), unilaminar primary follicle (UPF), antral follicle (AF), antrum (A), atretic follicle (yellow star), oocyte cytoplasm (white arrows), theca interna (TI), theca eksterna (TE), corpus luteum (CL), stromal connective tissue (white star), hemosiderin accumulation (yellow arrows), and lymphocyte infiltration (green arrows).

Healthy follicles at different developmental stages, corpus luteum, and a small number of atretic follicles were observed in the ovarian cortex of the control group. All components of connective tissue under the germinal epithelium and the medulla were in normal histological structure (Fig. 1A and B). In group 2, it was observed that the corpus luteum structures had increased considerably and the cortex was almost filled with new and regressed corpus luteum structures. The primordial follicles located under the germinal epithelium were mostly normal structures, but almost all follicles from the monolayer primary follicle were in atretic form and some of them had hemosiderin accumulation (Fig. 1C and D). Cystic formations, increased hemosiderin accumulation in connective tissue, atretic follicles, and corpus luteum were observed in group 3 as well. Additionally, diffuse lymphocyte infiltration was quite common below the germinal epithelium, and cortex and medulla in groups 2 and 3 (Fig. 1E and F).

### Follicle counting findings

It was observed that the numbers of primordial follicles were statistically significantly decreased in group 3 compared to the control (*P* < 0.05). No statistically significant differences in the number of primordial follicles were found between group 1 and group 2 (*P* = 0.136) and group 2 and group 3 (*P* = 0.198). The number of total primary follicles showed a statistically significant increase in groups 2 and 3 compared to the control (*P* < 0.05). However, the number of multilaminar atretic primary follicles was significantly increased in group 3 compared to the control (*P* < 0.05). When the total number of secondary follicles was evaluated, there was no significant difference between the groups (*P* = 0.077). Statistically significant decrease in the number of healthy antral follicles was found in groups 2 and 3 (*P* < 0.05). However, when the number of atretic antral follicles was evaluated, it was seen that this number was significantly lower in the control group compared to group 3 (*P* < 0.05). The number of Graaf follicles was significantly decreased in group 3 compared to the control (*P* < 0.05). No statistically significant differences in the number of Graaf follicles were found between groups 1 and 2 (*P* = 0.002) and groups 2 and 3 (*P* = 0.347). The number of cystic formation was statistically significantly increased in groups 2 and 3 compared to the control (*P* < 0.05). In addition, the corpus luteum formations were mostly found in group 2 compared to groups 1 and 3. In addition, the corpus luteum formations were mostly found in group 2 compared to groups 1 and 3. The results are shown in Table 1.

**Table 1:** Comparison of follicular count in all groups.

	Group 1 (*n* = 6)		Group 2 (*n* = 6)		Group 3 (*n* = 6)		*P* value
	Mean ± s.d.	Median (min–max)	Mean ± s.d.	Median (min–max)	Mean ± s.d.	Median (min–max)	
Primordial follicle	1967.83 ± 96.28	1963.00 (1806.00–2090.00)	1243.33 ± 79.68	1221.00 (1144.00–1354.00)	1060.16 ± 67.07	1034.50 (1002.00–1165.00)	0.001^b^
Unilaminar primary follicle	274.66 ± 46.81	254.00 (232.00–346.00)	453.33 ± 47.86	488.00 (369.00–520.00)	515.16 ± 56.29	511.50 (453.00–589.00)	<0.001^a^
Multilaminar healthy primary follicle	71.66 ± 5.81	69.00 (66.00–79.00)	73.16 ± 5,60	74.00 (64.00–80.00)	75.66 ± 5.31	74.50 (47.00–85.00)	0.474^a^
Multilaminar atretic primary follicle	2.33 ± 0.52	2.00 (2.00–3.00)	12.33 ± 1.03	12.00 (11.00–14.00)	15.66 ± 1.03	16.00 (14.00–17.00)	<0.001^b^
Healty antral follicle	50.16 ± 3.06	50 (46.00–54.00)	70.50 ± 8.09	72.50 (60.00–87.00)	67.33 ± 5.98	68.50 (58.00–73.00)	<0.001^a^
Atretic antral follicle	2.33 ± 5,51	2.00 (2.00–3.00)	9.6 ± 2.33	9.50 (6.00–13.00)	14.00 ± 1.78	14.50 (11.00–16.00)	0.001^b^
Graaf follicle	3.16 ± 0.75	3.00 (2.00–4.00)	1.50 ± 0.54	1.50 (1.00–2.00)	0.83 ± 0.75	1.00 (0.00–2.00)	<0.001^a^
Cyst	2.16 ± 0.75	2.00 (1.00–3.00)	7.83 ± 1.16	8.00 (6.00–9.00)	11.16 ± 1.60	11.00 (9.00–13.00)	<0.001^a^

^a^One-way ANOVA, ^b^Kruskal–Wallis test.

### IHC findings

#### PTEN findings

**Figure 2:**
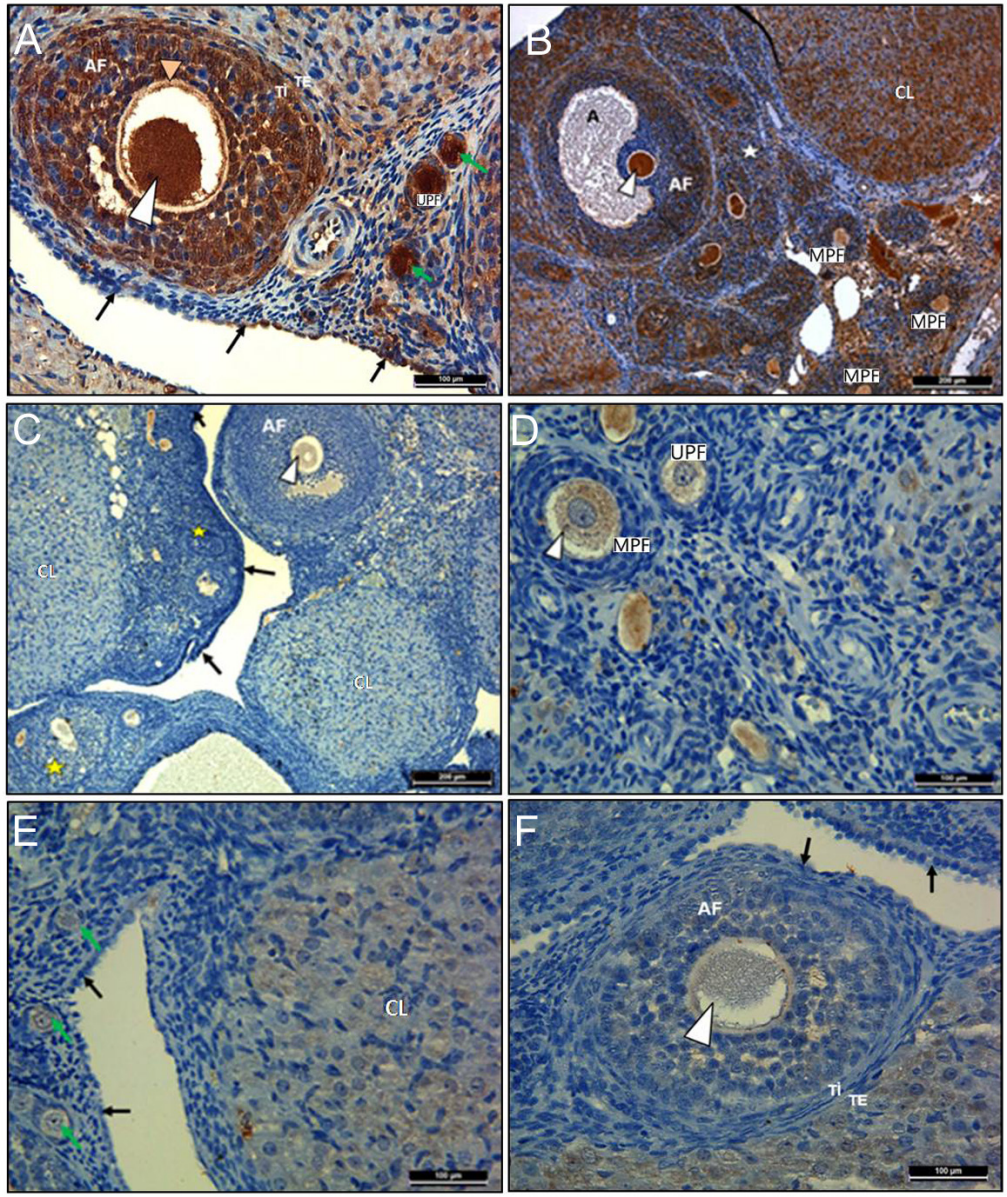
Results of immunohistochemical staining with PTEN primary antibody of ovarian tissues. Primordial follicles (green arrows), unilaminar primary follicle (UPF), multilaminar primary follicle (MPF), antral follicle (AF), antrum (A), atretic follicle (yellow star), oocyte cytoplasm (white arrows), theca interna (TI), theca eksterna (TE), and corpus luteum (CL).

**Figure 3:**
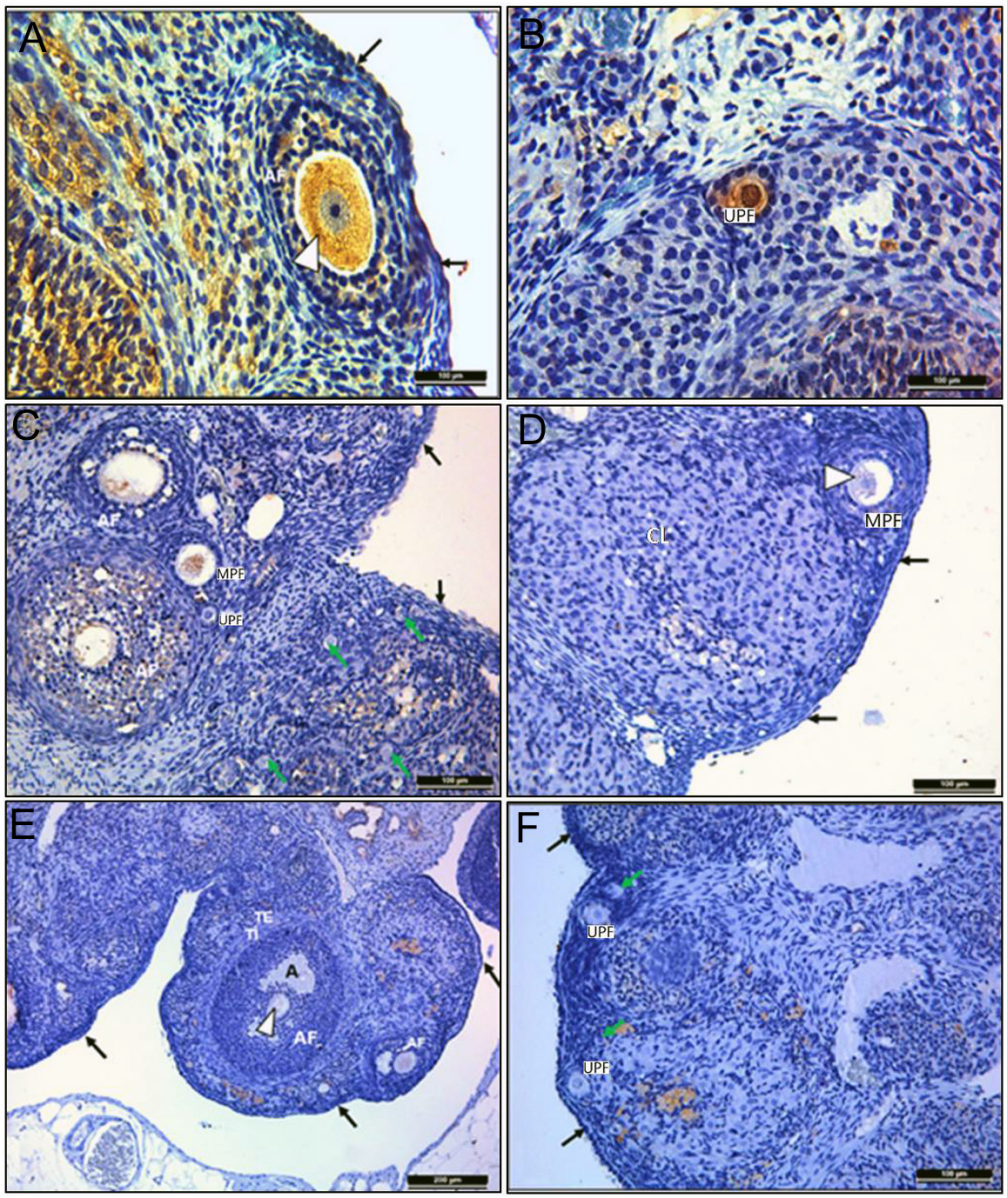
Results of immunohistochemical staining with FOXO3 primary antibody of ovarian tissues. Primordial follicles (green arrows), unilaminar primary follicle (UPF), multilaminar primary follicle (MPF), antral follicle (AF), antrum (A), atretic follicle (yellow star), oocyte cytoplasm (white arrows), theca interna (TI), theca eksterna (TE), and corpus luteum (CL).

The immunoreactivity levels of PTEN and FOXO3 in primordial follicles and LH-R in antral and Graaf follicles were evaluated in all groups using the H-Score method and compared statistically. The results are shown in Table 2. PTEN immunoreactivity was evaluated in the oocytes, granulosa cells, and theca cells of the follicles at every stage of follicular development. In the control group, as the fo∅llicles grew, PTEN expression increas∯ed in granulosa c≛ells but decreased in the≼ oocyte cytoplasm (Fig. 2A and B). Compared with the control group, PTEN expression was signi≼ficantly decreased in the oocyte∀ cytoplasm at every stage of follicular development in groups 2 (Fig. 2C and D) and 3 (Fig. 2E and F). Also, it almost disappeared in granulosa cells. The differences between groups 1 and 2 (*P* < 0.05), and groups 1 and 3 (*P* < 0.05) were statistically significant. This finding was consistent with the decreasing number of primordial follicles in groups 2 and 3. Additionally, there were no statistically significant differences in PTEN expression when groups 2 and 3 were compared (*P* = 0.162).

**Table 2:** Comparison of PTEN, FOXO3, and LH-R expression in all groups.

	Group 1 (*n* = 6)		Group 2 (*n* = 6)		Group 3 (*n* = 6)		*P* value
	Mean ± s.d.	Median (min–max)	Mean ± s.d.	Median (min–max)	Mean ± s.d.	Median (min–max)	
PTEN	2.77 ± 0.42	3.00 (2.00–3.00)	1.36 ± 0.68	1.00 (000–2.00)	0.94 ± 0.58	1.00 (0.00–2.00)	<0.001^b^
FOXO3	2.77 ± 0.43	3.00 (2.00–3.00)	1.61 ± 0.68	2.00 (0.00–2.00)	0.94 0.58	1.00 (0.00–2.00)	<0.001^b^
LH-R	2.75 ± 0.43	3.00 (2.00–3.00)	0.88 ± 0.66	1.00 (0.00–2.00)	0.41 ± 0.50	0.00 (0.00–1.00)	<0.001^b^

^b^Kruskal–Wallis test.

#### FOXO3 findings

The immunoreactivity of FOXO3 was evaluated in the oocytes, granulosa cells, and theca cells of the follicles at every stage of follicular development. In the control group, FOXO3 expression was found to be strong in the oocyte belonging to the primordial follicle but weak in the granulosa cells of the follicle. As the follicles grew, FOXO3 expression increased in granulosa cells but decreased in the oocyte cytoplasm (Fig. 3A and B). Compared with the control group, FOXO3 expression was significantly decreased in the oocyte cytoplasm at every stage of follicular development in groups 2 (Fig. 3C and D) and 3 (Fig. 3E and F). Also, it almost disappeared in granulosa cells. The differences between groups 1 and 2 (*P* < 0.05), and groups 1 and group 3 (*P* < 0.05) were statistically significant. This finding was consistent with the amount of PTEN expression in the oocyte cytoplasm and the number of primordial follicles. Additionally, there were no statistically significant differences in FOXO3 expression when groups 2 and 3 were compared (*P* = 0.006).

#### LH-R findings

**Figure 4:**
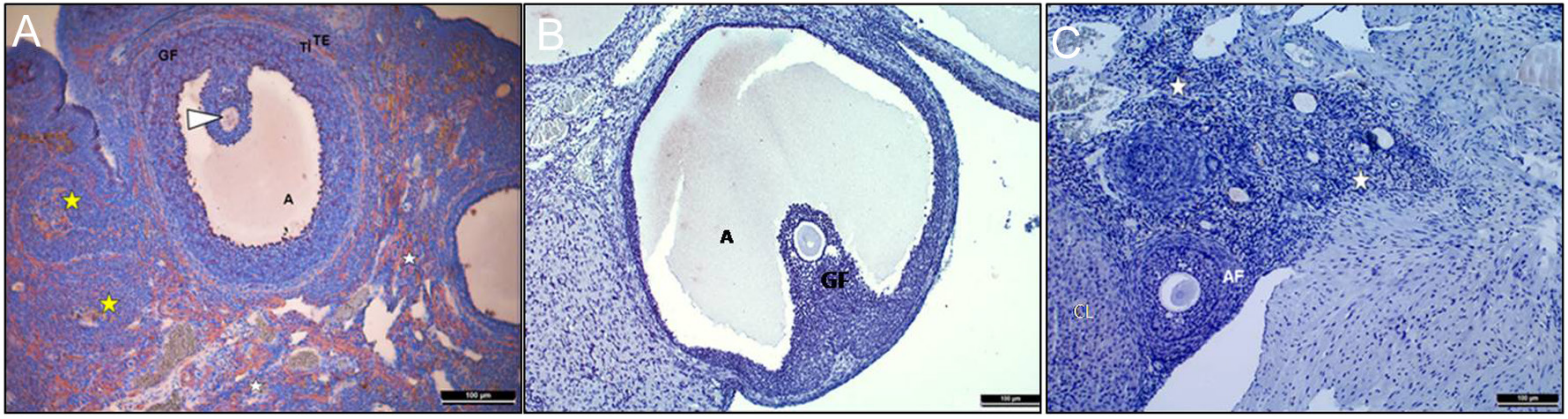
Results of immunohistochemical staining with LH-R primary antibody of ovarian tissues. Antral follicle (AF), Graaf follicle (GF), antrum (A), atretic follicle (yellow star), oocyte cytoplasm (white arrows), theca interna (TI), theca eksterna (TE), corpus luteum (CL), and stromal connective tissue (white star).

In the ovary, the immunoreactivity of LH-R, which is expressed in theca interna and granulosa cells of antral and Graaf follicles and corpus luteum structures, was evaluated in all experimental groups. In the control group, LH-R expression was found to be strong in theca interna and granulosa cells of antral and Graaf follicles (Fig. 4A). In contrast, it almost disappeared in theca interna and granulosa cell cytoplasm of Graaf follicles, antral follicles, and corpus luteum in groups 2 (Fig. 4B) and 3 (Fig. 4C). There were no statistically significant differences in LH-R expression in the oocyte cytoplasm when groups 2 and 3 were compared (*P* = 0.134). Whereas, the differences between groups 1 and 2 (*P* < 0.05), and groups 1 and 3 (*P* < 0.05) were statistically significant.

#### ELISA results

E2 level was observed to be increased in groups 2 and 3 compared to the control group, but the difference between groups was not statistically significant (*P* = 0.370 and *P* = 0.115, respectively). LH and FSH level was found to be increased in groups 2 and 3 compared to the control group, especially in group 2. Although the difference in LH level between control and group 3 was not statistically significant (*P*  = 0.976), the difference between groups 1 and 2 (*P* < 0.05) and groups 2 and 3 (*P* < 0.05) was statistically significant. Despite the difference in FSH level between control and group 2 was statistically significant (*P* < 0.05), there was no statistically significant difference observed between groups 1 and 3 (*P* = 0.864), and groups 2 and 3 (*P* = 0.148). All statistical results are shown in Table 3.

**Table 3:** Comparison of E2, LH, and FSH expression in all groups.

	Group 1 (*n* = 6)		Group 2 (*n* = 6)		Group 3 (*n* = 6)		*P* value
	Mean ± s.d.	Median (min–max)	Mean ± s.d.	Median (min–max)	Mean ± s.d.	Median (min–max)	
E2	60.00 ± 1.21	60.50 (42.00–5.00)	70.83 ± 1.14	69.00 (57.00–88.00)	74.83 ± 9.19	71.50 (67.00–90.00)	0.085^a^
LH	64.00 ± 1.61	64.00 (38.00–88.00)	97.66 ± 1.05	94.50 (88.00–110.00)	67.66 ± 1.73	67.00 (48.00–90.00)	0.003^a^
FSH	18.50 ± 5.99	19.50 (9.00–25.00)	42.50 ± 1.50	47.50 (18.00–56.00)	23.38 ± 1.50	23.00 (5.80–42.00)	0.013^a^

^a^One-way ANOVA

## Discussion

Some extracellular matrix components and growth factors that act in an autocrine-paracrine manner play role in the growth process of the ovarian follicles, which are the functional units of ovaries (Eppig 2001, Skinner 2005, Li *et al*. 2010, Pan* et al*. 2012). One of the most important mechanisms regarding primordial follicle activation is the PI3K signaling pathway (Hsueh *et al.* 2015). The molecules that are the components of this signaling pathway and which are considered to play roles in the regulation of follicle population dynamics are mainly PI3K, PIP2, PIP3, PDK-1, Akt, p27, TSC, mTOR, Foxo3a, and PTEN (Accili & Arden 2004, Kalich-Philosoph *et al*. 2013). PTEN, which is the negative regulator of the PI3K signaling pathway, works synergistically in coordination with the other molecules in this signaling pathway and suppresses the growth of primordial follicles protecting the ovarian reserve. Increased PTEN expression suppresses the expression of AKT, which plays role in many important pathways such as cell cycle, apoptosis, growth, and differentiation. In mammals, Akt inhibits p27, which is responsible for cell cycle arrest in the G1 phase and which is one of the most important suppressors in the transition from primordial follicle to the primary follicle, and early activation is thus seen in primordial follicles (Kiyokawa *et al*. 1996, Tong *et al*. 1998).

FOXO3 is a transcription factor expressed highly in the oocyte nucleus in primordial follicles playing roles in maintaining the ovarian reserve. In mice, it is known that decreased FOXO3 expression induces apoptosis in primordial follicles and reduces the ovarian reserve (Richards *et al*. 2002). In a similar study conducted with rodents, it was shown that oocyte-specific deletion of PTEN, Tsc1, and Foxo3a genes causes mass activation of dormant primordial follicles (Novella-Maestre *et al*. 2015). Although KOH is a technique that has been applied for years, no study has been found in the literature examining the effect of repeated KOH applications on ovarian reserve through the PI3K signal pathway. In line with these data, we hypothesized that the suppression mechanism of follicular growth may be impaired as one of the possible causes of primordial follicle loss that may occur in the ovarian reserve after KOH administration to rats in adulthood.

Therefore, in our study, we aimed to show possible changes in the expression of PTEN and FOXO3A, which act as suppressor molecules in the transition from primordial follicle to primary follicle, after applying KOH protocol methods, by immunohistochemical and biochemical methods.

When the study results were evaluated, PTEN and FOXO3 expressions were found to be higher in the control group compared to groups 2 and 3. Compared with the control group, PTEN and FOXO3 expression was significantly reduced in the oocyte cytoplasm at all stages of follicular development in groups 2 and 3. In addition, when groups 2 and 3 were compared, there was no statistically significant difference in PTEN and FOXO3 expression. In line with these data, we believe that decreased primordial follicle pool and expression of PTEN and FOXO3 molecules in our study were directly proportional in the groups that were treated with KOH. With the decrease of PTEN and FOXO3 expressions after KOH applications, the balance of the mechanism or mechanisms protecting the primordial follicle pool may be disturbed. The data of our study also support this situation.

Although the early stages of follicular development, which is a dynamic process, occur independently of gonadotropins, FSH and LH have very important effects on the selection of one single dominant follicle from the activated follicle group. For this reason, the interaction between gonadotropins and their receptors plays vital roles in the functioning of the female reproductive system (Yamoto *et al*. 1992, Zhang* et al*. 2001, Jeppesen *et al.* 2012). In humans, the expression of FSH-R is initiated at the multilayered primary follicle stage, on the surface of granulosa cells with the appearance of theca cells, and increases toward antral follicle (Minegishi *et al.* 1990, 1991, Zhang* et al*. 2001). FSH uses several intracellular signaling pathways, which include PI3K, to stimulate granulosa cells by interacting with FSH-R (Gonzalez-Robayna *et al*. 2000). In the literature, there are studies reporting that FSH activates PI3K signaling pathway during the follicular maturation process (Maizels *et al*. 1998, Alam *et al.* 2004) and any irregularity in this signaling pathway results in increased follicular atresia along with impaired steroid hormone synthesis (Chitnis *et al*. 2009). More than 100 target genes are activated, which play roles in the proliferation, growth, and differentiation of granulosa cells as a response to the mural granulosa cells that are stimulated by FSH. One of these genes is LH-R. Follicles must have a sufficient number of LH-Rs for a healthy progression to ovulation (Hobbs* et al*. 2012, Gong *et al*. 2019). It was emphasized in several studies in the past that LH-R expression is necessary for oocyte maturation, ovulation, and luteinization (Hunzicker-Dunn & Maizels 2006).

Zhang* et al*. in a study with LH-R knockout mice reported a delay in the formation of small ovaries, thin uterus, and vaginal opening in these mice compared to the control. In another study, it was reported that antral follicle formation was observed in the ovaries of LH-R knockout mice, but preovulatory follicle and KL formation were not observed. In this study, it was observed that follicular development did not go beyond the antral follicle even when the follicles were stimulated with high doses of FSH, and it was emphasized that LH-R is indispensable for follicular maturation (Yu *et al.* 2005, Hunzicker-Dunn & Maizels 2006).

In our study, it was also observed that LH-R expression was reduced at significant levels in the groups that were administered repeated KOH compared to the controls. We believe that this decrease in LH-R expression might have occurred because of the dysregulation caused by KOH administration in PIK3 signaling pathway. The findings such as decreased PTEN and FOXO3 molecule expressions, which are the main components of the PIK3 signaling pathway, obtained in our study, also support this.

In studies reported in the literature, increased E2 and FSH levels on the third day of the menstrual cycle are reported to indicate poor ovarian reserve (Mukherjee *et al.* 1996, Chuang *et al*. 2003). In our study, it was also found that the FSH, LH, and E2 levels were found to have increased in the groups that were treated with KOH compared to the controls. These data obtained in the study were also consistent with the findings of decreased primordial follicle number and decreased PTEN, FOXO3, and LH-R expression in these groups.

As a conclusion, it was determined in the present study that repeated COH reduced the expression levels of PTEN and FOXO3 proteins, which are known to protect the ovarian reserve as the main components of PIK3 intracellular signaling pathway, affect the levels of hormones such as FSH, E2, and LH, which are ovarian reserve markers, and has negative effects on the histological structure of the ovary, oocyte morphology, and count. In the light of all these data obtained here, we believe that multiple and consecutive applications of KOH protocols might decrease the primordial follicle pool, which is the indicator of ovarian reserve and might bring the age of menopause forward.

## Declaration of interest

The authors declare that there is no conflict of interest that could be perceived as prejudicing the impartiality of the research reported.

## Funding

This work was supported by the Gazi University Scientific Research Project (01/2017-14).

## Author contribution statement

P S conducted the experiments, statistical analysis and wrote the manuscript. C E provided guidance and assisted with manuscript editing. F Y Performed gene expression and statistical analyses.
